# The activation of PPARγ by 2,4,6-Octatrienoic acid protects human keratinocytes from UVR-induced damages

**DOI:** 10.1038/s41598-017-09578-3

**Published:** 2017-08-23

**Authors:** Enrica Flori, Arianna Mastrofrancesco, Daniela Kovacs, Barbara Bellei, Stefania Briganti, Vittoria Maresca, Giorgia Cardinali, Mauro Picardo

**Affiliations:** Laboratory of Cutaneous Physiopathology and Integrated Center of Metabolomics Research, San Gallicano Dermatologic Institute, IRCCS, Rome, Italy

## Abstract

Increasing attention is addressed to identify products able to enhance skin photoprotection and to prevent skin carcinogenesis. Several studies have demonstrated that the α-melanocyte stimulating hormone (αMSH), acting on a functional MC1R, provides a photoprotective effect by inducing pigmentation, antioxidants and DNA repair. We discovered a link between αMSH and the nuclear receptor Peroxisome Proliferator-Activated Receptor-γ (PPARγ), suggesting that some of the αMSH protective effects may be dependent on PPARγ transcriptional activity. Moreover, we demonstrated that the activation of PPARγ by the parrodiene 2,4,6-octatrienoic acid (Octa) induces melanogenesis and antioxidant defence in human melanocytes and counteracts senescence-like phenotype in human fibroblasts. In this study, we demonstrate that the activation of PPARγ by Octa exerts a protective effect against UVA- and UVB-induced damage on normal human keratinocytes (NHKs), the major target cells of UV radiation. Octa promotes the antioxidant defence, augments DNA repair and reduces the induction of proteins involved in UV-induced DNA damage response. Our results contribute to deepen the analysis of the αMSH/PPARγ connection and suggest perspectives for the development of new molecules and formulations able to prevent cutaneous UV damage by acting on the different skin cell populations through PPARγ activation.

## Introduction

Solar ultraviolet (UVR) radiation is the main aetiological agent of most types of skin cancer and a key factor responsible for photoaging^1, 2^. Over the past decade, there has been an increasing understanding on the mechanism by which UVR damage the skin. UVB can induce both direct and indirect adverse biological effects including DNA damage, oxidative stress, inflammation and immunosuppression. In addition, UVB can act as a tumour initiator, tumour promoter and co-carcinogen. On the other hand, the exposure to UVA induces the generation of reactive oxygen species (ROS), that can cause oxidative damage to proteins, lipids and DNA, leading to degeneration of dermal matrix, photo-aging, immunosuppression and photocarcinogenesis^1, 2^.

The skin expresses a highly regulated stress response system, equivalent to the hypothalamic-pituitary-adrenal (HPA) axis, to deal with many external biological or environmental factors and to maintain local and global homeostasis. This cutaneous neuroendocrine system is represented by the corticotrophin releasing hormone (CRH)/pituitary-derived propiomelanocortin (POMC) peptides system, capable of coordinating local responses to stress. The skin is a known target organ for the POMC-derived neuropeptides αMSH, β-endorphin, adrenocorticotropic hormone (ACTH) as well as a source of these peptides^3–5^.

Pigmentation, a coordinated enzymatic and non-enzymatic antioxidant defense network and DNA repair mechanisms provide endogenous efficient protection against UV radiation^6–8^. However, when the assault is too high, the innate defense systems become insufficient. Hence, there is increased attention to identify products capable of enhancing skin photoprotection and preventing skin carcinogenesis.

Depending on the skin cell types, different POMC-derived peptides are produced that act on specific melanocortin receptors (MC-Rs). Thus, processing of POMC and generation of the POMC-derived peptides is likely to be a key regulatory event in the skin^9, 10^. The key role of αMSH and its receptor MC1R in the induction of a protective response in melanocytes, which go beyond melanogenesis regulation, has been well established. An extensive and recent literature describes the ability of αMSH to induce antioxidants and DNA repair mechanisms, anti-inflammatory effects, protection from apoptosis, also in non-melanocytic populations, such as keratinocytes, fibroblasts, sebocytes and leukocytes^7, 8, 11–16^. These protective mechanisms are greatly reduced in cells expressing loss-of-function (LOF) MC1R^17–22^.

Surprisingly, many of the protective effects modulated by αMSH overlap with functions mediated by the pharmacological activation of the nuclear receptor Peroxisome Proliferator-Activated Receptor-gamma (PPARγ) (e.g. promotion of pigmentation and/or antioxidant and DNA repair systems)^23–26^. We have discovered the existence of a connection between αMSH and PPARγ in melanocytic cells through the induction of the PLC lipid involving-pathway. This link exerts an influence on melanogenesis and proliferation, suggesting its involvement in some of the extra-melanogenic effects afforded by the αMSH/MC1R interaction^27^.

PPARs are ligand-activated nuclear hormone receptors that require heterodimerization with the retinoid X receptor (RXR) for transcriptional activity. They regulate the expression of target genes containing PPAR-responsive elements in their promoter. Three isoforms of PPAR have been identified, PPARα, PPARβ/δ and PPARγ, all of which are expressed in human and mouse epidermis. Although the three PPAR isoforms exert overlapping functional roles, they exhibit distinct ligand binding specificity^28, 29^. In the skin, PPARs and their ligands have been shown to regulate important cellular functions, including cell proliferation and differentiation, as well as anti-inflammatory responses^29–32^. In particular, when activated by natural or pharmacological agonists, PPARγ has a crucial role in the control of cell differentiation, pigmentation, epidermal lipid synthesis, permeability barrier homeostasis, and containment of both inflammation and oxidative stress damage^23–26, 33, 34^.

We demonstrated that the activation of PPARγ by the parrodiene 2,4,6-octatrienoic acid (Octa) induces melanogenesis and antioxidant defence in normal human melanocytes *in situ* and *in vitro* ^35^. Moreover, in a stress-induced cellular senescence model^36^, we observed that PPARγ activation by Octa increases cell antioxidant defence and counteracts a senescence-like phenotype in primary cultures of human fibroblasts^37^.

Parrodienes, sharing some structural features with carotenoids and retinoids, are congeners of psittacofulvin, a class of pigments found in the red plumage of *Ara Macao*, serving as thermoregulation and defence against UV radiation^38^. Parrodienes possess antioxidant and anti-inflammatory activities and inhibit lipoperoxidation of cell membranes^39^.

Considering the beneficial role of PPARγ observed on primary melanocytes and fibroblasts, we evaluated whether the activation of PPARγ by the parrodiene Octa could exert a protective effects on UV-induced damage in normal human keratinocytes (NHKs), the major target cells of UV radiation.

The results demonstrated the capability of Octa to protect NHKs from UVA and UVB-damage, via PPARγ activation, resulting in increased cell survival and reduced apoptosis. Our data suggest perspective for the development of new formulations acting as complete sunscreen by targeting PPARs in the skin.

## Results

### Octa induces PPAR-γ expression and activation in NHKs

**Figure 1:**
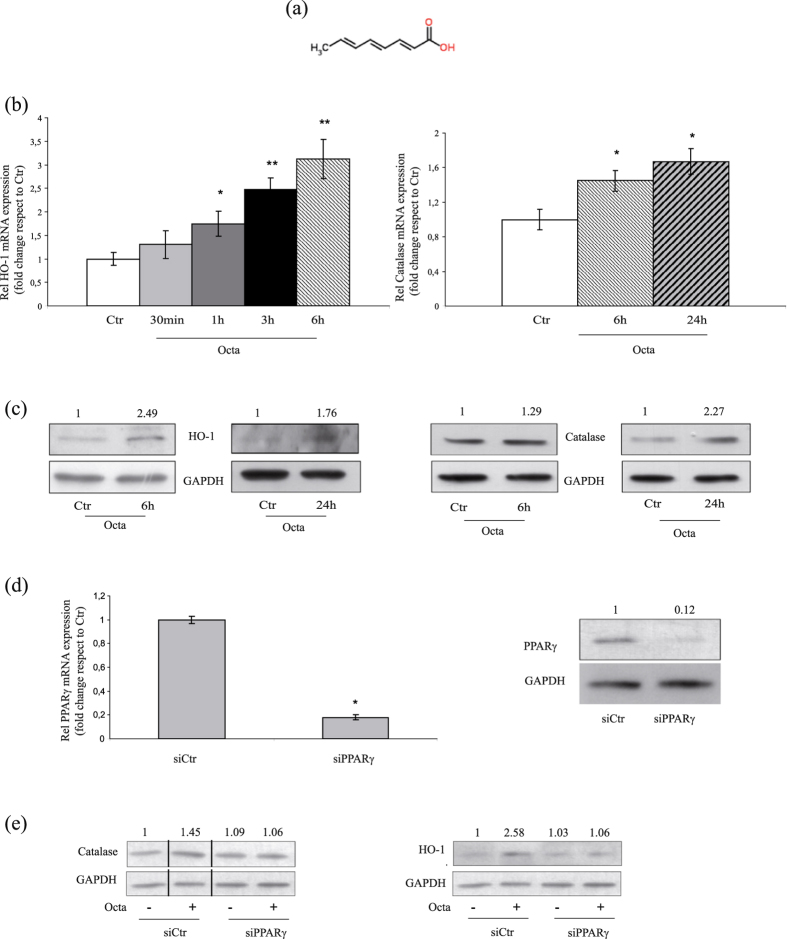
Octa stimulates the cellular antioxidant defense in NHKs through PPARγ activation. (**a**) Chemical structure of Octa (**b**) Expression of *HO-1* and *CATALASE* mRNA evaluated by quantitative real-time RT-PCR after 30 min, 1, 3, 6 and 24 h of treatment with 90 µM Octa, respectively. Values are normalized against the expression of glyceraldeide-3-phosphate dehydrogenase (*GAPDH*) and are expressed relative to untreated control cells. The values reported represent means ± SD of three independent experiments performed in triplicate. *p < 0,05, **p < 0,01 (vs untreated cells) (**c**) Western blot analysis of HO-1 and catalase protein expression on cell lysate of NHKs treated with 90 µM Octa for 6 and 24 h. GAPDH was used as an equal loading control. Results refer to three independent experiments. Representative blots are shown. Densitometric scanning of band intensities was performed to quantify the change of protein expression (control value taken as one fold in each case). (**d**) NHKs were transfected with siRNA specific for PPARγ (siPPARγ) or non-specific siRNA (siCtr). PPARγ level was evaluated by real-time RT-PCR (the values reported represent means ± SD of three independent experiments performed in triplicate. *p < 0,001, vs siCtr cells) and Western blot analysis using an anti-PPARγ antibody. Results refer to three independent experiments. Representative blots are shown. Densitometric scanning of band intensities was performed to quantify the change of protein expression (control value taken as one fold). (**e**) Western blot analysis of HO-1 and catalase protein expression after 24 h exposure to 90 µM Octa in NHKs transfected with siPPARγ and siCtr. GAPDH was used as a loading control. Results refer to three independent experiments. Representative blots are shown. Densitometric scanning of band intensities was performed to quantify the change of protein expression (control value taken as one fold in each case).

We initially verified the ability of Octa (chemical structure showed in Fig. 1a) to activate PPARγ in keratinocytes. Time course qRT-PCR showed that the exposure to Octa induced a rapid increase (30 minutes, 1 h) of PPARγ mRNA (Supplementary Fig. 1a). A luciferase assay using pGL3-(Jwt)TKLuc reporter construct^40^ consistently demonstrated an enhanced luciferase expression soon after a 30 minute treatment of Octa (Supplementary Fig. 1b). Parallel Western Blot analysis confirmed an increase in PPARγ protein level after Octa treatment (Supplementary Fig. 1c). These data indicate the ability of the compound to induce both PPARγ expression and activity in keratinocytes.

### Octa stimulates the cellular antioxidant defense in NHKs through PPARγ activation

We previously observed that Octa induces cell antioxidant defense in normal human melanocytes^35^ and fibroblasts^37^. Therefore we investigated whether Octa would also induce the antioxidant defence system in NHKs. The treatment with Octa determined an early up-regulation, both at mRNA (Fig. 1b) and protein levels (Fig. 1c), of HO-1 and catalase, two key enzymes in cellular defence against oxidative stress. This induction was not due to an intracellular ROS increase following treatment with Octa (Supplementary Fig. 2a). Considering that PPARγ regulates the expression of catalase and HO-1 via functional PPREs identified in their promoter^25, 26^, we examined whether the enhanced cellular antioxidant defence observed in Octa-treated NHKs was specifically due to PPARγ activation. To this end, we evaluated catalase and HO-1 protein expression in NHKs transiently transfected with siRNA for PPARγ (siPPARγ) or control (siCtr) (Fig. 1d). As expected, Octa significantly increased catalase and HO-1 expression in siCtr cells but failed to induce them in PPARγ-deficient NHKs (Fig. 1e). The presence of GW9662, a specific and selective PPARγ inhibitor^41^, corroborated these results (Supplementary Fig. 2b). The results conclusively document the critical involvement of PPARγ in the Octa-mediated activation of the cellular antioxidant defence in NHKs.

### Octa pre-treatment protects NHKs from UVA and UVB-induced cell death and apoptosis

**Figure 2:**
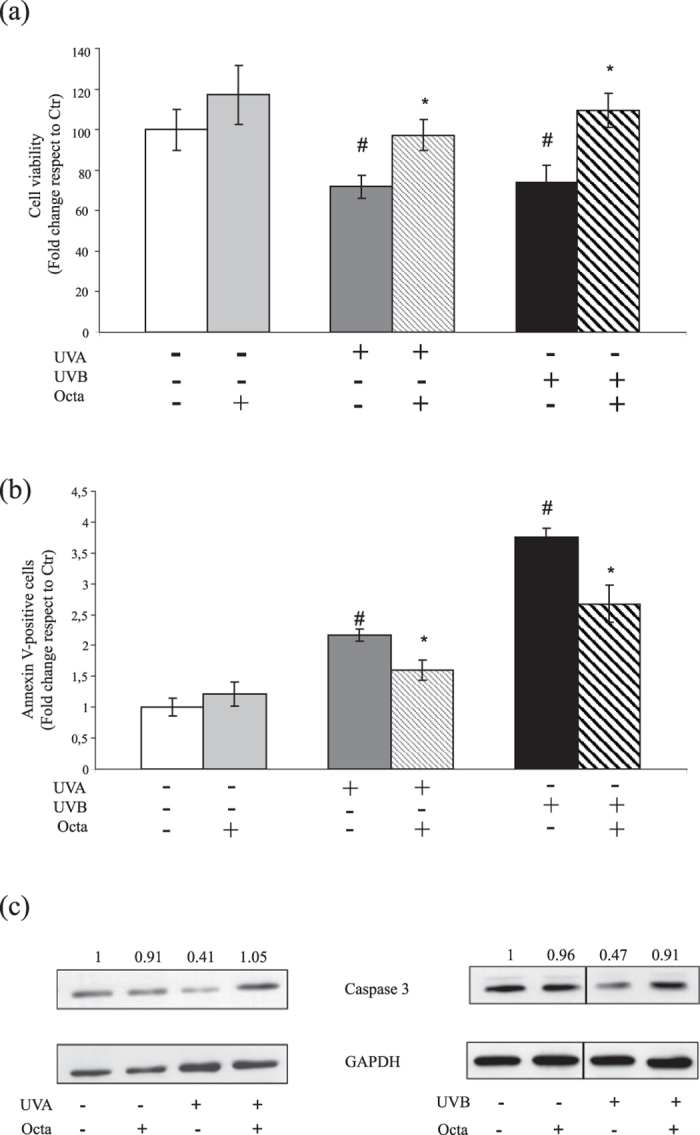
Octa pre-treatment protects NHKs from UVA and UVB-induced cell death and apoptosis. (**a**) NHKs were irradiated with UVA 10 J/cm2 or UVB 25 mJ/cm2 after a 24 h pre-treatment with 90 µM Octa. Viability was evaluated by Neutral Red assay 48 h after irradiation. The values reported represent means ± SD of three independent experiments performed in quadruplicate. ^#^p < 0,005 (vs untreated cells), *p < 0,01 (vs UV-irradiated cells) (**b**) NHKs were pre-incubated with 90 µM Octa for 24 hrs and irradiated with UVA 10 J/cm2 or UVB 25 mJ/cm2. Apoptosis was evaluated by FACS analysis 16 h after irradiation, using annexin V labelling. The values reported represent means ± SD of three independent experiments performed in duplicate. ^#^p < 0,005 (vs untreated cells), *p < 0,01 (vs UV-irradiated cells) (**c**) NHKs were pre-treated with 90 µM Octa for 24 h and then irradiated with UVA 10 J/cm2 or UVB 25 mJ/cm2. Proteins were extracted 16 h later and Western blot analysis of caspase 3 protein expression was performed. GAPDH was used as a loading control. Results refer to three independent experiments. Representative blots are shown. Densitometric scanning of band intensities was performed to quantify the change of protein expression (control value taken as one fold in each case).

Based on the above results, we investigated whether Octa may have a cytoprotective effect against the damage induced by UVA and UVB exposure. NHKs were exposed to UVA (10 J/cm2) or UVB (25 mJ/cm2) and then allowed to recover for 48 h. UVA or UVB alone induced a significant decrease in cell viability compared to control, whereas the pre-treatment with Octa for 24 h provided substantial protection, as measured by Neutral Red assay (Fig. 2a). To test the protective properties of Octa against UVA- and UVB-mediated apoptosis, we also examined the modification of the apoptosis marker annexin V by FACS analysis. Octa treatment significantly reduced the amount of annexin V-positive NHKs at 16 h compared to that observed after UVA or UVB alone (Fig. 2b). Western blot analysis consistently showed the activation of caspase 3, a critical executioner of apoptosis following UVA or UVB, as assessed by the decrease in the protein expression of the inactive zymogen, and pre-treatment with Octa counteracted this effect (Fig. 2c). The results demonstrated that PPARγ activation increased cell survival and lowered apoptosis following UVA and UVB.

### Octa pre-treatment protects NHKs from UVA and UVB-induced DNA damage

**Figure 3:**
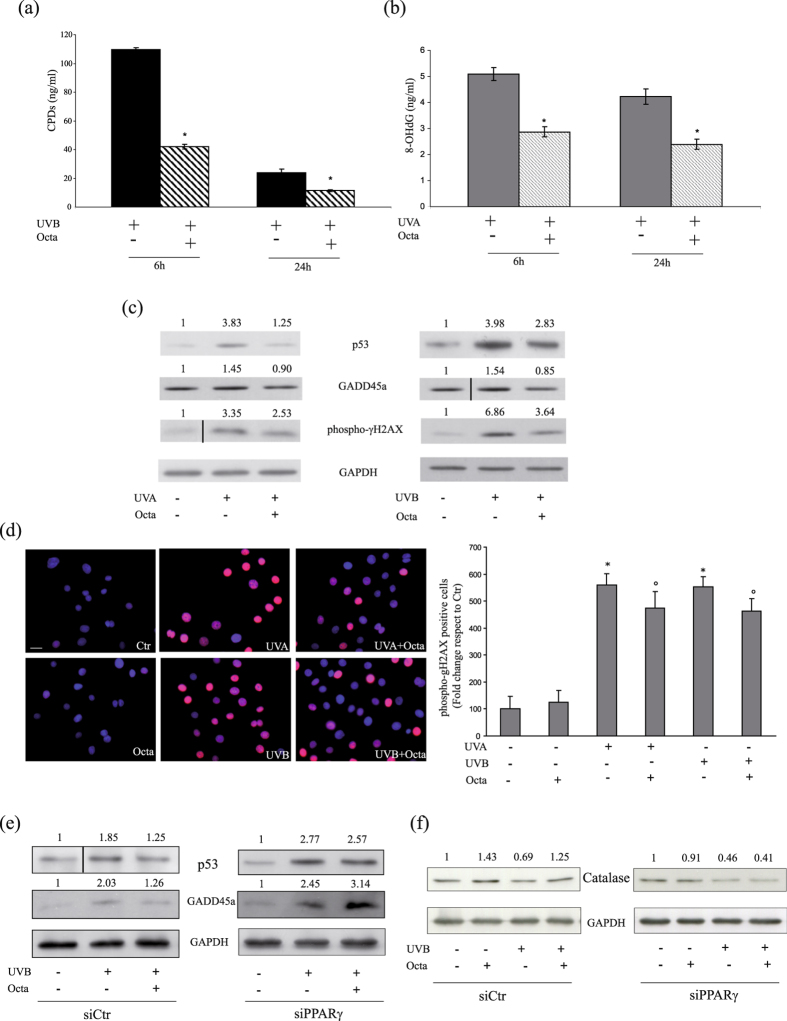
Octa protects NHKs from UVA and UVB-induced DNA damage, through PPARγ activation. ELISA analysis of the amount of CPDs (**a**) and 8-oxodG (**b**) remaining in NHKs pre-treated with 90 µM Octa for 24 h and irradiated with UVA 10 J/cm2 or UVB 25 mJ/cm2. DNA was extracted 6 and 24 h later. The values reported represent means ± SD of three independent experiments performed in duplicate. *p < 0,001 (vs UVB-irradiated cells), *p < 0,005 (vs UVA-irradiated cells) (**c**) NHKs were pre-incubated with 90 µM Octa for 24 h and then irradiated with UVA 10 J/cm2 or UVB 25 mJ/cm2. Proteins were extracted 24 h later and Western blot analysis of p53, GADD45a and phospho-γH2AX protein expression was perfomed. GAPDH was used as a loading control. Results refer to three independent experiments. Representative blots are shown. Densitometric scanning of band intensities was performed to quantify the change of protein expression (control value taken as one fold in each case). (**d**) Immunofluorescence analysis of phosphorylated histone H2AX expression (red signal) in NHKs pre-treated with 90 µM Octa for 24 h and irradiated with UVA 10 J/cm2 or UVB 25 mJ/cm2. Corresponding quantitative analysis of the fluorescence signal evaluated by counting the number of positive cells/total cells. Nuclei are counterstained with DAPI (blue). Scale bar: 20 µm. The values reported represent means ± SD of two distinct experiments. *p < 0,001 (vs non-irradiated cells), °p < 0,005 (vs UV-irradiated cells). Western blot analysis of p53, GADD45a (**e**) and catalase (**f**) protein expression using lysates obtained from NHKs transfected with siPPARγ and siCtr, exposed for 24 h to 90 µM Octa and irradiated with UVA 10 J/cm2 or UVB 25 mJ/cm2. Proteins were extracted 24 h later. GAPDH was used as a loading control. Results refer to three independent experiments. Representative blots are shown.

DNA is a key molecular-target for the deleterious effects of UV. To investigate whether the cytoprotective effect of Octa correlates with reduction of DNA lesions, we analysed cyclobutane-pyrimidine dimmers (CPDs) and 7,8-dihydro-8-oxoguanine (8-oxodG), two major DNA damage induced by UVB and UVA. Soon after the 6 h following irradiation, the amount of CPDs (Fig. 3a) and 8-oxodG (Fig. 3b), evaluated by ELISA assay, were markedly decreased in Octa-pre-treated cells compared to cells exposed to UVB or UVA alone, suggesting that Octa accelerated DNA repair. The tumour suppressor p53 and its target gene GADD45a (growth-arrest and DNA damage gene) are critical factors in the protection of the epidermis against UV-induced tumourigenesis promoting apoptosis and/or cell cycle arrest and DNA repair of damaged keratinocytes, thus preventing the expansion of mutant or deregulated cells^42^. The analysis of p53 and GADD45a expression in NHKs exposed to UVA or UVB displayed that cells pre-treated with Octa are less damaged by UVA or UVB, as demonstrated by the reduced levels of both markers (Fig. 3c). We also analysed the expression levels of phosphorylated histone H2A.X (γ-H2A.X), the expression of which is associated with the amount of DNA double strand breaks after UV-radiation^43, 44^. After UVA and UVB irradiation, NHKs pre-treated with Octa showed a reduced expression of this UV-related genotoxicity marker by Western blot analysis (Fig. 3c). These results were further confirmed by immunofluorescence experiments. Upon exposure to both UVA and UVB an increase in the number of NHKs stained for phosphorylated histone H2A.X was observed. Octa pre-treatment significantly reduced the number of positive cells compared with the staining of irradiated NHKs alone (Fig. 3d). To investigate whether the protective effects observed in Octa-treated NHKs could be specifically due to PPARγ activation, we evaluated p53 and GADD45a protein expression in siPPARγ-NHKs or siCtr-cells after UVB exposure. Octa significantly counteracted the induction of both markers in siCtr cells but failed to prevent their up-regulation in PPARγ-deficient NHKs (Fig. 3e). Moreover, Octa significantly induced the expression of catalase in siCtr-NHKs, both in basal conditions and in response to UVB, whereas it failed to up-regulate the antioxidant enzyme in PPARγ-deficient NHKs (Fig. 3f). All together, these results underline the ability of Octa to protect NHKs from UVA and UVB-induced DNA damage and document the critical involvement of PPARγ in this biological activity.

### Photoprotective effect of Octa post-treatment on UVA and UVB-induced DNA damage in NHKs

**Figure 4:**
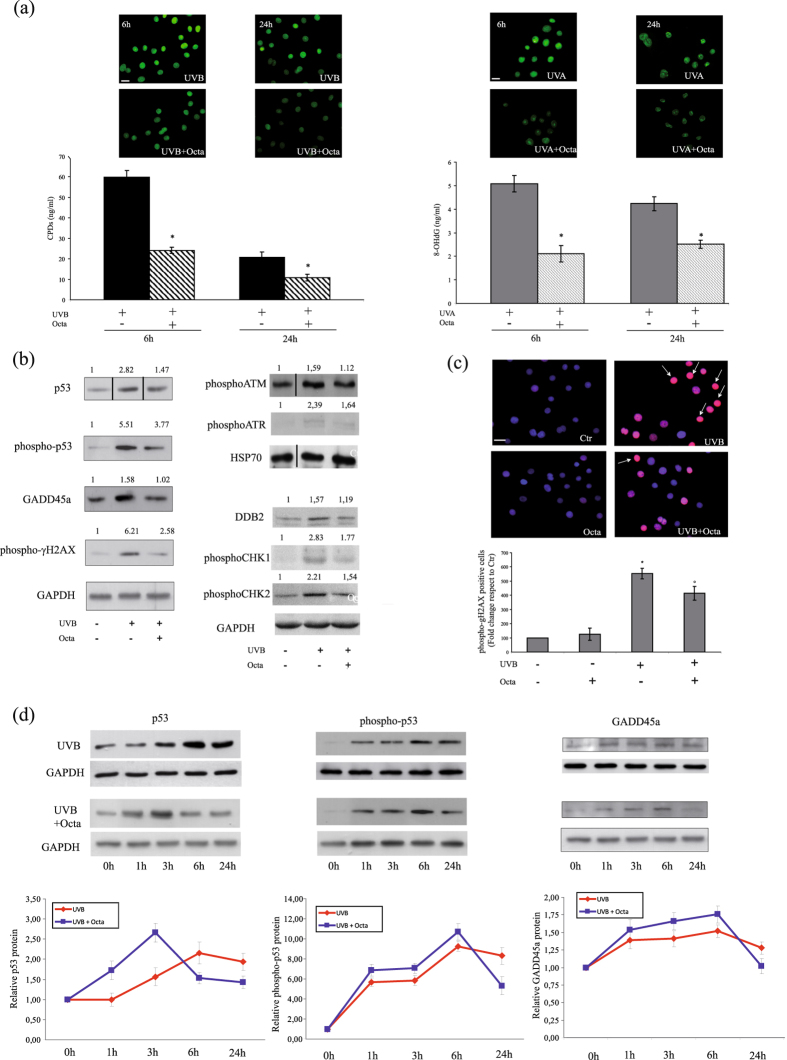
Effect of Octa post-treatment on UVB-induced DNA damage. (**a**) Immunofluorescence analysis of CPDs and 8-oxodG (green signal) in NHKs irradiated with UVB 25 mJ/cm2 or UVA 10 J/cm2 and post-treated with 90 µM Octa for 6 h and 24 h. Scale bar: 20 µm. ELISA analysis of the amount of CPDs and 8-oxodG remaining in NHKs irradiated with UVB 25 mJ/cm2 or UVA 10 J/cm2 and post-treated with 90 µM Octa for 6 and 24 h. The values reported represent means ± SD of three independent experiments performed in duplicate. *p < 0,001 (vs UVB-irradiated cells), *p < 0,005 (vs UVA-irradiated cells). (**b**) Western blot analysis of p53, phospho-p53, GADD45a, phospho-γH2AX, phosho-ATM, phospho-ATR, DDB-2, phospho-Chk1 and phosphor-Chk2 protein expression on cell lysate of NHKs irradiated with UVB 25 mJ/cm2 and post-treated with 90 µM Octa for 24 h. GAPDH and HSP70 were used as a loading control. Results refer to three independent experiments. Representative blots are shown. Densitometric scanning of band intensities was performed to quantify the change of protein expression (control value taken as one fold in each case). (**c**) Immunofluorescence analysis of phosphorylated histone H2AX expression (red signal) in NHKs irradiated with UVB 25 mJ/cm2 and post-treated with 90 µM Octa for 24 h. Arrows indicate super-bright positive cells which results reduced in Octa post-treated cells respect to UVB irradiated cells. Quantitative analysis of the phosphorylated histone H2AX fluorescence signal evaluated by counting the number of positive cells/total cells. Nuclei are counterstained with DAPI (blue). Scale bar: 20 µm. The values reported represent means ± SD of two distinct experiments. *p < 0,001 (vs non-irradiated cells), °p < 0,005 (vs UVB-irradiated cells) (**d**) Time course analysis of the effect of 90 µM Octa post-incubation on the protein expression levels of p53, phospho-p53 and GADD45a in UVB (25 mJ/cm2)-exposed NHKs. GAPDH was used as a loading control. Results refer to three independent experiments. Representative blots are shown.

We also performed some experiments adding the compound soon after UV-irradiation to confirm that, besides its UV-absorbing properties, Octa also exerts photoprotective effects through cellular activities. Octa post-treatment displayed a photoprotective effect similar to that exerted by the pre-treatment as regards cell death and apoptosis. In fact, following UVA or UVB exposure, Octa post-incubation enhanced the overall cell viability (Supplementary Fig. 3a) and significantly reduced the amount of annexin V-positive NHKs (Supplementary Fig. 3b). Even the amount of CPDs and 8-oxodG, evaluated by ELISA and immunofluorescence analysis, was markedly decreased in Octa post-treated cells compared to NHKs exposed to UVB or UVA alone (Fig. 4a), confirming the ability of the molecule to accelerate DNA repair. At 6 h post irradiation, the number of CPDs positive cells was higher in UVB treated cells compared to cells post-treated with Octa (Fig. 4a). At 24 h post irradiation, there was a reduction in the amount of positive cells with respect to that observed at 6 h due to the activation of DNA repair systems^45, 46^. Octa post treatment was still effective in protecting the cells against the damaging effects of UVB irradiation. We further deepened the investigation of the protective effect of Octa post-incubation in NHKs irradiated with UVB. In addition to the expression γ-H2A.X, p53, phospho-p53 and GADD45a, we analyzed the following protein levels: (a) the nucleotide excision repair (NER) initiation factor UV-damaged DNA-binding protein 2 (DDB-2), which is capable of recognizing CPD lesions and to bind avidly to UV-irradiated DNA^47^; (b) phospho-ATM, the activation of which occurs in response to NER-mediated DNA double strand break by autophosphorylation on Ser1981; (c) phospho-ATR (Ser428), the primary sensor of single-stranded breaks caused by UV damage; (d) phospho-Chk1 (Ser345) and phospho-Chk2 (Thr68), ATM/ATR substrates, as well as p53, which play an important role in DNA damage checkpoint control, delaying cells progression through the cell cycle^47, 48^. Western blot analysis demonstrated that 24 h Octa post-incubation decreased the levels of the proteins involved in DNA damage response in UVB-irradiated NHKs (Fig. 4b). The reduction in the γ-H2A.X expression was also evaluated by immunofluorescence analysis, which confirmed the ability of Octa post-treatment to significantly reduce the number of γ-H2A.X positive keratinocytes induced following exposure to UVB (Fig. 4c). Moreover, as previously described^43^, the γH2AX immunostaining identifies the presence of two different population of positive cells following UVB irradiation: bright and super-bright cells, which are associated with cell cycle arrest/DNA repair and apoptosis conditions, respectively. The percentage of γH2AX super-bright positive cells/total positive cells results lower in keratinocytes post-treated with Octa respect to UVB irradiated cells, confirming the protective role of the molecule (Ctr: 2.78 ± 08%; Octa: 2.22 ± 0.66%; UVB: 46.42 ± 2.64%; UVB + Octa: 38.1 ± 3.59%). Finally, the time course analysis indicated that p53, phospho-p53 and GADD45a proteins levels continued to increase steadily for up to 24 h in cells exposed to UVB. Octa post-incubation of the UVB-exposed cells, however, accelerated the increase in the expression levels of these proteins at earlier times, peaking at 3 h for p53 and 6 h for phospho-p53 and GADD45a, before gradually declining thereafter (Fig. 4d). These results indicated that post-treated NHKs recover from the deleterious effect of UVB exposure more efficiently than the untreated counterpart does. All together, these results clearly demonstrated that Octa operates not only as a chemical filter but also through active biological mechanisms.

### Differences in photoprotective effects between Octa and αMSH in WT or MC1R loss of function NHKs

**Figure 5:**
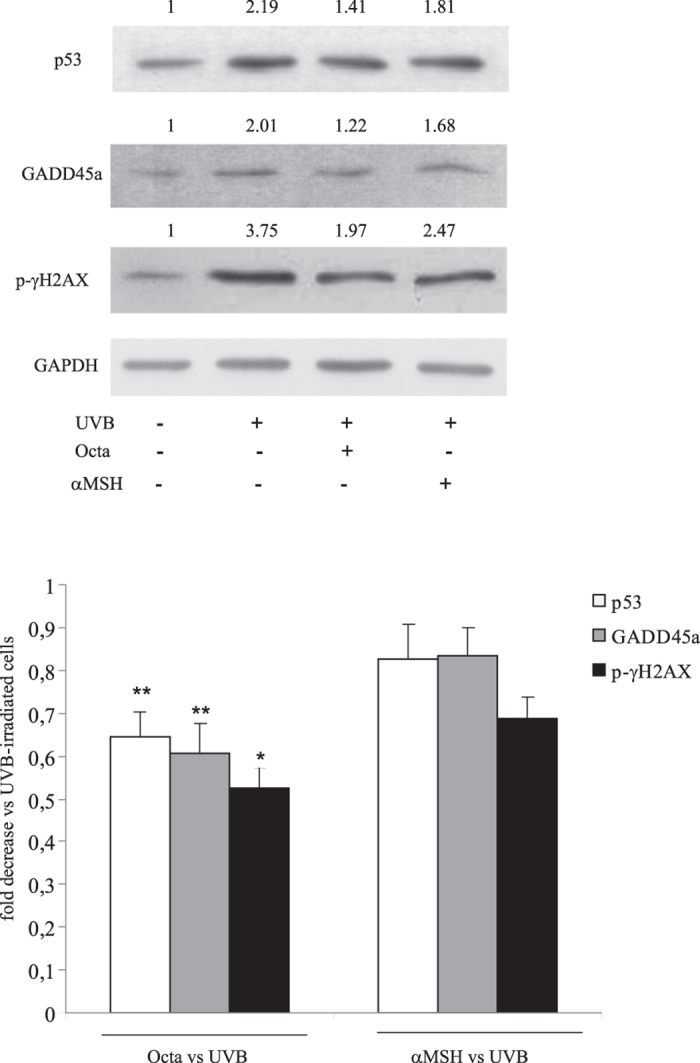
Photoprotective effect of Octa or αMSH in WT or *MC1R* loss of function NHKs. Western blot analysis of p53, GADD45a and phospho-γH2AX protein expression on cell lysate of NHKs, WT or LOF (Arg_151_Cys, Arg_160_Trp, Asp_84_Glu) for *MC1R*, pre-treated with 90 µM Octa or 10^−7^M αMSH for 24 h and irradiated with UVB 25 mJ/cm2. Proteins were extracted 24 h later. GAPDH was used as a loading control. Representative blots are shown. Densitometric scanning of band intensities was performed to quantify the change of protein expression (control value taken as one fold in each case). The values reported represent means ± SD of three independent experiments. *p < 0,05 (vs αMSH-treated UVB-irradiated cells), **p < 0,01 (vs αMSH-treated UVB-irradiated cells).

Non-melanoma skin cancers (NMSC) are the most common malignancies in fair-skinned populations with a continuing increase in incidence during recent decades^22, 49^. Data in literature demonstrate that the risk of NMSC development is higher among carriers of *MC1R* variants, especially of LOF *MC1R* variants^18, 22^. We asked whether the activation of PPARγ by Octa could provide photoprotective effects in cells harbouring inactivating mutations of *MC1R*. We selected NHK heterozygous cultures for LOF *MC1R* variants (Arg_151_Cys, Arg_160_Trp, Asp_84_Glu) with the aim of comparing the protective activity of Octa and αMSH. We analyzed the effect of αMSH or Octa on the expression of p53, GADD45a and γ-H2A.X in response to UVB irradiation. Western blot analysis revealed that Octa was more effective than αMSH in decreasing the levels of the proteins involved in DNA damage response in UVB-irradiated LOF NHKs (Fig. 5), suggesting that Octa also retained its protective effect in cells carrying *MC1R* variants.

### Photoprotective effects of Octa pre- and post-treatment on human epidermal skin equivalents exposed to UVB

**Figure 6:**
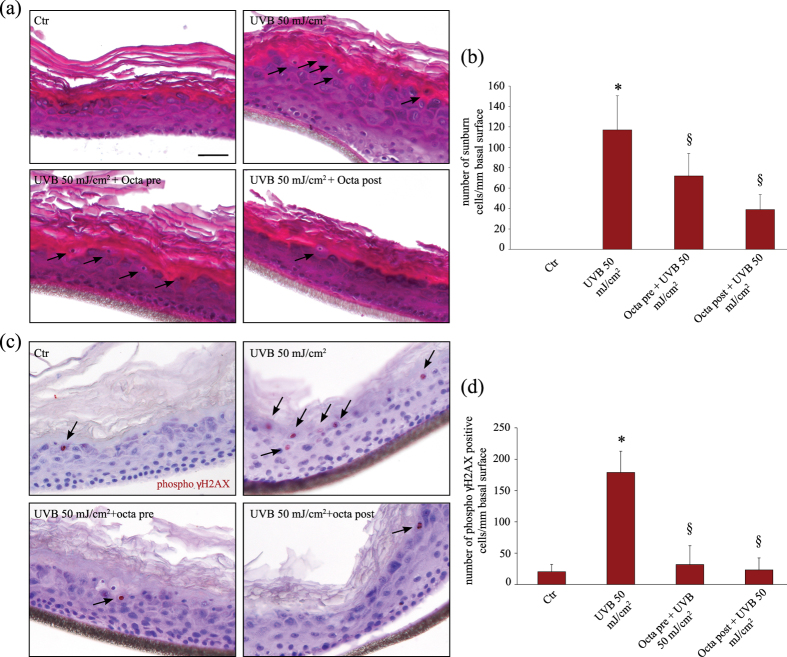
Photoprotective effect of Octa in epidermal equivalents. (**a**) Haematoxylin-eosin staining of skin samples exposed to UVB 50 mJ/cm2 and pre or post-treated with 90 µM Octa. Arrows point sunburn cells. Nuclei are counterstained with haematoxylin. Scale bar: 50 µm. (**b**) Quantitative analysis of the number of sunburn cells expressed as mean value ± SD per mm of basal surface. *p < 0,01 (vs non-irradiated cells), ^§^p < 0,01 (vs UVB-irradiated cells) (**c**) Immunohistochemical analysis of phospho-γH2AX positive cells (arrows) of skin samples exposed to UVB 50 mJ/cm2 and pre or post-treated with 90 µM Octa. Nuclei are counterstained with haematoxylin. Scale bar: 50 µm. (**d**) Corresponding quantitative analysis of the signal evaluated by counting the number of positive cells/total cells. *p < 0,01 (vs non-irradiated cells), ^§^p < 0,01 (vs UVB-irradiated cells).

To validate our *in vitro* results we next analysed the effects of pre- and post-treatment with Octa on human epidermal skin equivalents exposed to UVB. We first evaluated the formation of sunburn cells (SBCs) as an index of cells undergoing apoptosis in response to acute UVB injury^50, 51^. Skin equivalents exposed to UVB (50 mJ/cm2) showed a significant increase in the number of SBCs respect to non irradiated control. The treatment with Octa, applied either before or after the irradiation, caused a significant reduction of SBCs (p < 0.01) (Fig. 6a,b). Human epidermal skin equivalents exposed to UVB were then evaluated for the expression of the phosphorylated histone H2A.X. UVB irradiation significantly increased the number of stained cells, similarly to that observed in the *in vitro* experiments, and Octa pre- treatments and Octa post-treatments were able to reduce the amount of γ-H2A.X positive cells (p < 0,01) (Fig. 6a,d), counteracting the DNA damage induced by UV exposure.

## Discussion

Despite numerous prevention campaigns and growing importance of photoprotective measures, the incidence of skin cancer continues to rise dramatically^22, 49^. In addition to physical barriers, including clothing and sunglasses, topical sunscreens filtering UVB and UVA spectrum remain one of the most widely adopted strategies against photo-damage^52, 53^. There is an intense research aimed at identifying new products able to increase endogenous defence of the skin. In this regard, the protective benefit derived from incorporating antioxidants in sunscreens to replenish the natural reservoirs has been demonstrated in human studies^54, 55^.

The present findings document the critical involvement of PPARγ in mediating the protection of NHKs from UVA and UVB-induced apoptosis and DNA damage, as well as the induction of the antioxidant defence. Our results indicate that PPARγ specific modulators, such as Octa, are endowed with good skin photoprotective properties.

The ability to protect the skin against UV insult or ROS-induced DNA damage has been demonstrated for other molecules originating from natural resources and endowed with antioxidant and cytoprotective activity, such as green tea polyphenols, silymarin, soy isoflavones and resveratrol^1, 2, 56^. For instance, the flavonoid afzelin inhibits the UVB-mediated increase in lipid peroxidation, the formation of CPDs and cell death in human keratinocytes, with a combination of UV-absorbing and active cellular activities^57^. When administered in combination, genistein and daidzein flavonoids exert a synergistic photoprotective action against inflammation and DNA damage induced by UVB^58^. Green tea polyphenols reduce UVB-induced CPDs in normal human keratinocytes and human skin equivalents^59, 60^.

Octa deserves to be fully explored as a novel candidate skin photo-protectant for its relevant features: (i) it is free from long-term side effects, as demonstrated both *in vitro* and *in vivo* ^61, 62^; (ii) it simultaneously exerts its beneficial effects in the three main skin cell populations, suggesting perspectives for its development as a molecule with a complete sunscreen efficacy; (iii) it promotes multiple facets of endogenous skin protection, through biological mechanism of action involving PPARγ activation. The post-treatment data and PPARγ silencing experiments added important insights into the action mechanism of Octa, demonstrating that the compound operates non only as a chemical filter but even through active biological mechanisms (e.g reduction of oxidative damages and induction of DNA repair mechanisms). p53 expression in keratinocytes is generally accepted as a reasonable indicator of DNA damage degree^63^. A certain threshold of p53 phosphorylation on Ser15 in UVR-irradiated cells might separate cell cycle arrest/DNA repair from apoptosis commitment^64^. Moreover, the correlation between UVB-induced phosphorylation of H2AX and p53 has been demonstrated, suggesting that the presence of super-bright γH2AX cells could be an early indicator that the threshold of p53 phosphorylation which separates cell cycle arrest/DNA repair from apoptosis commitment has been reached^43^. In UVB-irradiated NHKs post-treated with Octa, we observed an accelerated increase in p53 at earlier time frames accompanied by its quickened phosphorylation, which concurs to its accumulation and activation^65^. This accelerated increase in p53, speeding up the expression of its target genes involved in DNA repair mechanisms and ROS containment, may contribute to the faster repair of UVB photoproducts and the consequent reduction of the observed levels of the proteins involved in DNA damage cascade response following UVB damage, starting from the initiation factor DDB-2, the protein sensors (ATM/ATR), the signal transducers, e.g. Chk1 and Chk2, and the effectors, as p53 and GADD45a. These results were confirmed by the observation that UVB-irradiated NHKs post-treated with Octa showed not only a decreased total expression of γ-H2AX respect to UVB-irradiated cells, but also a lower percentage of super-bright positive cells, representing cells which have arrested the cell cycle to repair the DNA damage^43^. Furthermore, PPARγ silencing was able to completely abrogate the protective effect of Octa, clearly indicating that the activation of PPARγ is needed for the biological effect mediated by the molecule.

**Figure 7:**
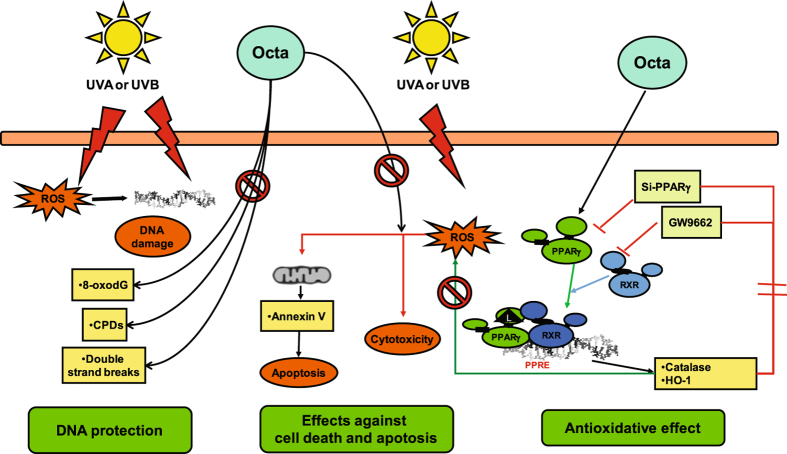
Summary scheme of possible role of PPARγ activation in counteracting UV-induced damage. The activation of PPARγ by Octa exerts a protective effect against UVA- and UVB-induced damage on normal human keratinocytes, the major target cells of UV radiation. Octa promotes the antioxidant defence and counteracts the UV-induced DNA damage and apoptosis.

In skin, PPARγ regulates the expression of genes involved in multiple biological pathways^28–31, 33, 34^. However, limited studies examine the role of PPARs in photobiology. Some data show that PPARγ induces differentiation in response to natural or pharmacological agonists, reproducing some of the effects promoted by αMSH in melanocytes and melanoma cells^23–26^. We demonstrate that the activation of PPARγ by Octa induces melanogenesis and antioxidant defense in normal human melanocytes^35^ and counteracts photo-induced cell senescence in primary cultures of human fibroblasts^37^. The study by Sahu *et al*. provides evidence that epidermal PPARγ plays a protective role in suppressing UVB-induced tumour formation and progression in mice^66^. The beneficial effects of a novel PPARα/γ dual agonist on inflammatory responses, epidermal thickness and lipid peroxidation associated with chronic UVB exposure have also been demonstrated^31^. Moreover, Wang *et al*.^67^ show that mice lacking the PPARγ heterodimerization partner RXRα in epidermal keratinocytes exhibit increased apoptosis, altered epidermal proliferation as well as increased DNA damage in response to UVB exposure. The present findings, demonstrating the protective effect of PPARγ activation against UVA and UVB-induced damage on the main target cells of UV radiation, reinforced the evidence that the activation of PPARs by natural or pharmacological agonists could afford photoprotective effects (Fig. 7).

The key role αMSH and its wild type receptor MC1R play in the induction of extra-melanogenic protective responses, such as the induction of antioxidants and DNA repair mechanisms, in melanocytes and non-melanocytic populations, is well known. MC1R expression is not restricted to melanocytes and has been observed in several other cell types including keratinocytes, fibroblasts, monocytes, dendritic and endothelial cells^6–8, 11–15^. Moreover, important new findings link the MC1R to nuclear receptors activation, shedding light on some of the extra-melanogenic effects dependent on αMSH activity. The induction of NR4A subfamily of nuclear receptors by MC1R activation in melanocytic cells seems to be a key component of MC1R-mediated DNA repair following UVR^68^ and the αMSH/PPARγ connection we discovered in melanocytic cells exerts an influence on melanogenesis and proliferation^8, 27^.

The defensive mechanisms exerted by αMSH are greatly reduced in cells expressing a loss of function *MC1R*. Some of these variants are closely associated with the red hair and fair skin phenotype, a condition highly linked to the development of melanoma and keratinocyte-derived skin cancer^17–20, 22^. Data in literature indicate that *MC1R* variants reduce capacity for *in vitro* ^21^ and *in vivo* ^69^ DNA repair. Furthermore, LOF *MC1R* variants confer lower protection against UVR-induced ROS in fibroblasts and keratinocytes^20^. Consistent with MC1R’s role in photo-protection, *MC1R* variants, particularly LOF *MC1R* variants, are associated with a strongly increased risk of severe photo-aging^70^. The striking results we obtained on LOF NHKs documented the potential of PPARγ activation in providing photoprotective effects in cells harbouring inactivating mutations of *MC1R*. The ability to reproduce the beneficial effects afforded by αMSH, through the activation of nuclear receptors, would represent an important protective strategy against UV radiation in fair-skinned individuals.

In conclusion, this study contributes to deepen the analysis of the αMSH/PPARγ connection, the understanding of which might shed light on some of the beneficial extra-melanogenic effects dependent on the αMSH/MC1R interaction and new ways through which such functions are modulated. Moreover, the strategy to develop new molecules and formulations acting on the different skin cell populations through PPARγ is expected to be beneficial in preventing cutaneous UV damage and cancer, by augmenting DNA repair and antioxidant defence, and stimulating melanogenesis, particularly in high-risk individuals, e.g. those harbouring MC1R mutations.

## Material and Methods

### Cell culture and treatments

Primary cultures of human keratinocytes (NHKs) were isolated from neonatal foreskin in accordance with a previously described procedure^71^. NHKs were grown at 37 °C under 5% CO_2_ in the defined medium M154 (Invitrogen Life Technologies Italia, Monza, Italy) with Human keratinocyte growth supplements (HKGS) (Invitrogen Life Technologies Italia, Monza, Italy). Keratinocytes were sub-cultured once a week and experiments carried out in cells between passage 2 to 4. The Institute’s Research Ethics Committee (IFO) approval was obtained to collect samples of human material for research. The study was conducted according to the Declaration of Helsinki Principles. Patients gave written informed consent. For each experiment at least three different donors were used. Cells were plated and 24 h later were stimulated with chemicals in fresh medium, in accordance with the experimental design. The following doses of chemicals were employed: 90 μM Octa (kindly supplied by Giuliani Pharma SpA, Milan, Italy), manufactured from 2,4-trans-hexadienal by a chemical process for synthesis which comprises four stages and is patented by Giuliani Pharma SpA; the purity grade of Octa assayed by HPLC-UV was 99,8%; 10^−7^M αMSH (Sigma-Aldrich Srl, Milan, Italy); 3 μM GW-9662 (Sigma-Aldrich Srl, Milan, Italy), a potent and irreversible antagonist of PPARγ^41^. For UV irradiation, NHKs were incubated in medium without phenol-red and irradiated at a dose of 10 J/cm2 for UVA and 25 mJ/cm2 for UVB using a Bio-Sun irradiation apparatus (Vilbert Lourmat, Marne-la-Vallée, France). Control cells were treated identically but without UV exposure. The UVA lamps in the illuminator emit ultraviolet rays between 355 nm and 375 nm, with peak luminosity at 365 nm; UVB lamps emit ultraviolet rays between 280 and 320 nm, with peak luminosity at 312 nm. Neither UVA nor UVB lamps have UVC emission. UVA and UVB were supplied by a closely spaced array of four UVA lamps or two UVB lamps which delivered uniform irradiation at a distance of 10 cm. Based on a programmable microprocessor, the Bio-Sun system constantly monitors the UV light emission. The irradiation stops automatically when the energy received matches the programmed energy (range of measure: 0 to 9,999 J/cm2).

### Reconstructed skin

The epidermal equivalent Epiderm was obtained from MatTek Corp. (Ashland, MA), using normal human keratinocytes obtained from Caucasian neonatal foreskin tissues. Epidermal equivalents were grown at the air/liquid interface of the maintenance medium EPI-100-MM (MatTek Corp). The epidermal equivalents were pre or post-treated with Octa, according to the experimental design, and irradiated at the dose of 50 mJ/cm2 UVB using a Bio-Sun irradiation apparatus (Vilbert Lourmat, Marne-la-Vallée, France).

### RNA extraction and quantitative real-time RT-PCR

Total RNA was isolated using the Aurum^TM^ Total RNA Mini kit (Bio-Rad Laboratories Srl, Milan, Italy). Following DNAse I treatment, cDNA was synthesized using oligo-dT primers and using ImProm-II^TM^ Reverse Transcriptase (Promega Corporation, Madison, WI) according to the manufacturer’s instructions. Quantitative real time RT-PCR was performed in a total volume of 15 μl with SYBR Green PCR Master Mix (Bio-Rad Laboratories Srl) and 200 nM concentration of each primer. The primer sequences were as follows: PPARγ sense: 5′-TCAAACGAGAGTCAGCCTTTAACG-3′ and antisense: 5′-AGTGGGAGTGGTCTTCCATTACG-3′; Catalase sense: 5′-TTTCCCAGGAAGATCCTGAC-3 and antisense: 5′-ACCTTGGTGAGATCGAATGG-3′, HO-1 sense: 5′-CCAGCGGGCCAGCAACAAAGTGC-3′ and antisense 5′-AAGCCTTCAGTGCCCACGGTAAGG-3′; GAPDH sense: 5′-TGCACCACCAACTGCTTAGC-3′ and antisense: 5′-GGCATGGACTGTGGTCATGAG-3′. Reactions were carried out in triplicates using the Real-Time Detection System iQ5 (Bio-Rad Laboratories Srl) supplied with iCycler IQ5 optical system software version 2.0 (Bio-Rad Laboratories Srl). Melt curve analysis was performed to confirm the specificity of the amplified products. Glyceraldehyde-3-phosphate dehydrogenase (GAPDH) was used as an endogenous control.

### Transient transfection and luciferase assay

NHKs (1 × 10^6^ cells) were transfected with 2.5 μg pGL3-(Jwt)3TKLuc reporter construct^40^ using Amaxa^®^ human keratinocyte Nucleofector kit (Lonza, Basel, Switzerland). A pTK-Renilla-expressing vector was also transfected as an internal control. After 24 h of transfection, cells were exposed to Octa (90 μM). After treatment, cells were harvested in 100 μl lysis buffer and 20 μl of the extract was assayed for luciferase activity using Promega’s Dual Luciferase (Promega Corporation, Madison, WI) according to the manufacturer’s protocol. The luciferase activity was expressed as a fold of the activity obtained in cells treated, divided by luciferase activity from non-stimulated cells.

### Western blot analysis

Cells were lysed in denaturing conditions supplemented with protease inhibitor cocktail (Roche, Mannheim, Germany). The protein concentration of extracts was estimated with Bradford reagent (Bio-Rad Laboratories Srl, Milan, Italy). Equal amounts of proteins were then separated on acrylammide SDS-PAGE, transferred onto nitrocellulose (Amersham Biosciences, Milan, Italy) and then treated overnight at 4 °C with anti-HO-1 antibody (1:1000; Enzo Life Sciences, Lausen, Switzerland); anti-Catalase antibody (1:1000; Sigma-Aldrich Srl, Milan, Italy); anti-Caspase 3 antibody, anti-GADD45a antibody, anti-phospho-γH2AX (Ser139) antibody, anti-DDB-2 antibody, anti-phospho-p53 (Ser15) antibody (all 1:1000; Cell Signalling Technology, New England Biolabs, UK); anti-phospho-ATM (Ser1981) antibody, anti-phospho-ATR (Ser428) antibody, anti-phospho-CHK1 (Ser345) antibody, anti-phospho-CHK2 (Thr68) antibody, (all 1:1000; DNA damage antibody sampler kit (#9947) Cell Signalling Technology); anti p53 antibody (1:1000; DAKO Cytomation, Glostrup, Denmark). HRP-conjugated goat anti-mouse (1:3000; Cell Signalling Technology) or anti-rabbit IgG (1:8000; Cell Signalling Technology) were used as secondary antibodies. Antibody complexes were visualized using the enhanced chemiluminescence reagent (ECL) (Amersham Biosciences, Milan, Italy). A subsequent hybridization with anti-GAPDH or anti-HSP70 (1:5000) (Santa Cruz Biotechnology Inc., Santa Cruz, CA, USA) were used to estimate the equal protein loading. Densitometric analysis was performed using a GS-800 Calibrated Image Densitometer (Bio-Rad Laboratories Srl, Milan, Italy).

### RNA interference experiments

For the RNA interference experiments, NHKs were transfected with 100 pmols (h) siRNA specific for PPARγ (sc-29455; Santa Cruz Biotechnology, Santa Cruz, CA, USA). An equivalent amount of non-specific siRNA (sc-44234; Santa Cruz Biotechnology, Santa Cruz, CA, USA) was used as a negative control. Cells were transfected using Amaxa^®^ human keratinocyte Nucleofector kit (Lonza, Basel, Switzerland), according to manufacturer’s instructions. To ensure identical siRNA efficiency among the plates, cells were transfected together in a single cuvette and plated immediately after nucleofection. 24 h following transfection, treatments were added to some samples in agreement with the experimental design.

### Neutral Red assay

At the end of the treatment, cells were incubated with Neutral Red (0.05 mg/ml) (Sigma Aldrich Srl) for 2 h at 37 °C and lysed in acetic acid/ethanol solution. The absorbance at 540 nm was measured by a spectrophotometer µQUANT (Biotek Instruments, Winooski, VT, USA).

### Apoptosis analysis

Cell death and apoptosis were detected by annexin V staining. Briefly, NHKs were harvested by trypsinization, resuspended in the staining buffer (10 mm HEPES ⁄ NaOH, pH 7.4, 140 mm NaCl, 2.5 mm CaCl2), stained with FITC-labelled annexin V and propidium iodide, 1 μg/ml each and then analyzed by FACSCalibur (Becton Dickinson, Mountain View, CA, USA). A total of 10 × 10^3^ cells cells from each sample were acquired, and CELL-QUEST software (Becton Dickinson) was used to analyze the data.

### ELISA assay

Genomic DNA from cell cultures was extracted using Tissue Kit (Qiagen, Milan, Italy). DNA samples were converted to single-stranded DNA by incubation at 95 °C for 10 min and rapidly chilling on ice for 10 min. CPDs and 8-OHdG were quantified with the Oxiselect UV-induced DNA damage ELISA combo kit and the Oxiselect Oxidative DNA damage ELISA kit, respectively (both from Cell Biolabs, San Diego, CA, USA), according to the manufacturer’s protocol.

### Immunofluorescence analysis

Cells grown on coverslips previously coated with 2% gelatine in PBS on 24-well plates. Following treatments with Octa and UV irradiation cells were fixed in 4% formaldehyde in PBS for 15 min at room temperature following the incubation in blocking buffer (5% goat serum, 0.3% Triton X-100 in PBS) for 1 h at room temperature. Cells were then incubated overnight at +4 °C with anti phospho-Histone H2A.X rabbit mAb (Cell Signaling Technology, #9718) (1:400 in 1% BSA, 0.3% Triton X-100 in PBS). The primary antibody was visualized with goat anti-rabbit Alexa Fluor 555 conjugate antibody (Cell Signaling Technology, #4413) (1:500 in 1% BSA, 0.3% Triton X-100 in PBS) after appropriate washing with PBS. For the detection of 8-oxo-dG cells were processed according to the protocol (Anti-8-oxo-dG, Clone 2E2 – Cat# 4354-MC-050 – Trevigen, Heigerman Court, Galthersburg, MD, USA). Nuclei were visualized using DAPI (Sigma-Aldrich Srl). Fluorescence signals were analyzed by recording stained images using a cooled CCD colour digital camera (Zeiss, Oberkochen, Germany). Quantitative analysis of the phospho-Histone H2A.X positive cells was performed evaluating at least 200 cells for each condition. Results are expressed as percentage of number of positive cells/total cells (mean value ± SD) relative to control cells. Positive cells with saturated nucleus were considered “super-bright” positive cells and the quantitative analysis was performed evaluating at least 200 cells for each condition. Results are expressed as percentage of number of super-bright positive cells/total positive cells (mean value ± SD). For CPDs detection cells were fixed in 4% PFA in PBS for 10 minutes at room temperature and processed according to the protocol (Anti-cyclobutane pyrimidine dimmers (CPDs) – Code n. NMDND001 – Cosmobio Co. LTD, Tokyo, Japan).

### Immunohistochemistry

Epidermal equivalents were formalin-fixed and paraffin-embedded (FFPE). Serial sections were dewaxed in xylene and rehydrated through graded ethanols to PBS, pH 7.4. For histological analysis sections were stained with haematoxylin and eosin. For immunohistochemical analysis endogenous peroxidase activities were blocked by 0.03% hydrogen peroxide (Genemed Biotechnologies, South San Francisco, CA, USA). Sections were incubated with anti-phospho-Histone H2A.X rabbit mAb (1:400 in PBS) (Cell Signaling Technology, New England Biolabs, UK; #9718) and the staining was visualized using Power-stain kit poly HRP employing 3-amino-9-ethyl-carbazole as substrate chromogen (Genemed). The sections were counterstained with haematoxylin antigen retrieval was achieved by heating sections in 10 mM citrate buffer, pH 6. For images analysis pictures were collected by CCD colour digital camera (Zeiss, Oberkochen, Germany). Sunburn cells and phospho-γH2A.X were counted along the whole section and were expressed as number of positive cells ± SD per mm of basal surface, calculated using the AxioVision 4.7.1 software (Zeiss).

### Genetic characterization of *MC1R*

Genomic DNA from cell cultures was extracted using Tissue Kit (Qiagen, Milan, Italy). About 3–5 μl of genomic DNA was subject to PCR in a total volume of 50 μl containing 25 μl of 2x PCR Master Mix (Promega, Madison, WI, USA) and 25 pmol of forward primer 5′-GGCAGCACCATGAACTAAGCAGG-3′ and reverse primer 5′-GGACCAGGGAGGTAAGGAAC-3′. DNA fragments were checked by electrophoresis in 2% agarose gel and purified using High Pure PCR Product Purification Kit (Roche Diagnostics GmbH, Mannheim, Germany). DNA fragments were sequenced in both strands by automated sequencing.

### Statistical analysis

Statistical significance was assessed using paired Student’s t-test. The minimal level of significance was P < 0.05.

### Data Availability

The datasets generated during and/or analyzed during the current study are available from the corresponding author on reasonable request.

## Electronic supplementary material

Supplementary Information
