# Supplementary material for: The activation of PPARγ by 2,4,6-Octatrienoic acid protects human keratinocytes from UVR-induced damages
Source: Sci Rep. 2017 Aug 23;7:9241. doi: 10.1038/s41598-017-09578-3 (PMC5569026; doi:10.1038/s41598-017-09578-3)
Supplement: Supplementary file 1 — Supplementary Information [file 41598_2017_9578_MOESM1_ESM.pdf]

Supplementary Information

The activation of PPARγ by 2,4,6-Octatrienoic acid protects human keratinocytes from UVR-induced damages

Enrica Flori[1], Arianna Mastrofrancesco[1], Daniela Kovacs[1], Barbara Bellei[1], Stefania Briganti[1], Vittoria Maresca[1], Giorgia Cardinali[1], and Mauro Picardo[1*]

All the authors are from: Laboratory of Cutaneous Physiopathology and Integrated Center of Metabolomics Research, San Gallicano Dermatologic Institute, IRCCS, Rome, Italy

[*]Mauro Picardo: Corresponding author

Supplementary Figure 1. The effect of Octa on PPARγ activation in NHKs. (**a**) Quantitative real-time RT-PCR was performed to measure the expression of *PPAR*γ mRNA after 30 min, 1, 3 and 6 h of treatment with 90 μM Octa. Values are normalized against the expression of glyceraldeide-3-phosphate dehydrogenase (*GAPDH*) and are expressed relative to untreated control cells. The values reported represent means ± SD of three independent experiments performed in triplicate . $^{*}p<0,01$ (vs untreated cells) (**b**) Transcriptional activity of PPARγ by luciferase activity analysis of cells transfected with pGL3-(Jwt)3TKLuc reporter construct. After 24 h of transfection, cells were treated with 90 μM Octa. The measurement of luciferase activity was carried out 30 min, 1, 3, 6 and 24 h after treatment. The variability of transfection was normalized with renilla luciferase activity. The results were expressed as fold change with respect to untreated cells. The values reported represent means ± SD of three independent experiments performed in duplicate. $^{*}p<0,05$ (vs untreated cells), $^{**}p<0,01$ (vs untreated cells) (**c**) Western blot analysis of PPARγ protein expression on cell lysate of NHKs treated with 90 μM Octa for 3, 6 and 24 h. GAPDH was used as equal loading control. Results refer to three independent experiments. Representative blots are shown. Densitometric scanning of band intensities was performed to quantify the change of protein expression (control value taken as onefold in each case).

Supplementary Figure 2. The effect of Octa on intracellular ROS content and catalase expression . (**a**) ROS production was evaluated by cytofluorimetric analysis using 2.5 μM DCFH-DA after 30 min, 1, 3, and 6 h of treatment with 90 μM Octa, respectively. Results refer to three independent experiments performed in duplicate (**b**) Western blot analysis of catalase protein expression on cell lysate of NHKs pre-treated for 1 h with GW-9662 and then treated with 90 μM Octa for 24 h. GAPDH was used as an equal loading control. Results refer to three independent experiments. Representative blots are shown. Densitometric scanning of band intensities was performed to quantify the change of protein expression (control value taken as onefold in each case).

Supplementary Figure 3. Effect of Octa post-treatment on UVA and UVB-induced cell death and apoptosis. (**a**) NHKs were irradiated with UVA 10 J/cm2 or UVB 25 mJ/cm2 and post-incubated for 48 h with 90 μM Octa. Viability was evaluated by Neutral Red assay. The values reported represent means ± SD of three independent experiments performed in quadruplicate. $^{\#}p<0,005$ (vs untreated cells), $^{*}p<0,01$ (vs UV-irradiated cells) (**b**) FACS analysis of apoptosis (annexin V labelling) in NHKs irradiated with UVA 10 J/cm2 or UVB 25 mJ/cm2 and post-treated

with 90 μM Octa for 16 h. The values reported represent means ± SD of three independent experiments performed in duplicate. #p<0,005 (vs untreated cells), *p<0,01 (vs UV-irradiated cells).

Supplementary Material and Methods

ROS detection

Immediately after treatment, NHKs were washed twice with PBS and incubated with 2.5 μM 2',7'-dichlorofluorescein diacetate (DCFH-DA) (Fluka AG, Buchs, Switzerland) in 154 medium without phenol red at 37°C and 5% CO_2. After 30 min, cells were detached, centrifuged and resuspended in 154 medium without phenol red. The oxidative conversion of DCFH-DA to the fluorescent product in living cells was assessed by flow cytometry, using a FACSCalibur (Becton Dickinson, Mountain View, CA, USA). A total of 10×10^3 cells cells from each sample were acquired, and CELL-QUEST software (Becton Dickinson) was used to analyse the data. The intracellular ROS were quantified using the median of FL-1 channel of fluorescence, because it matches the maximal number of cells with the highest fluorescence. The results represent the mean of three experiments in duplicate.

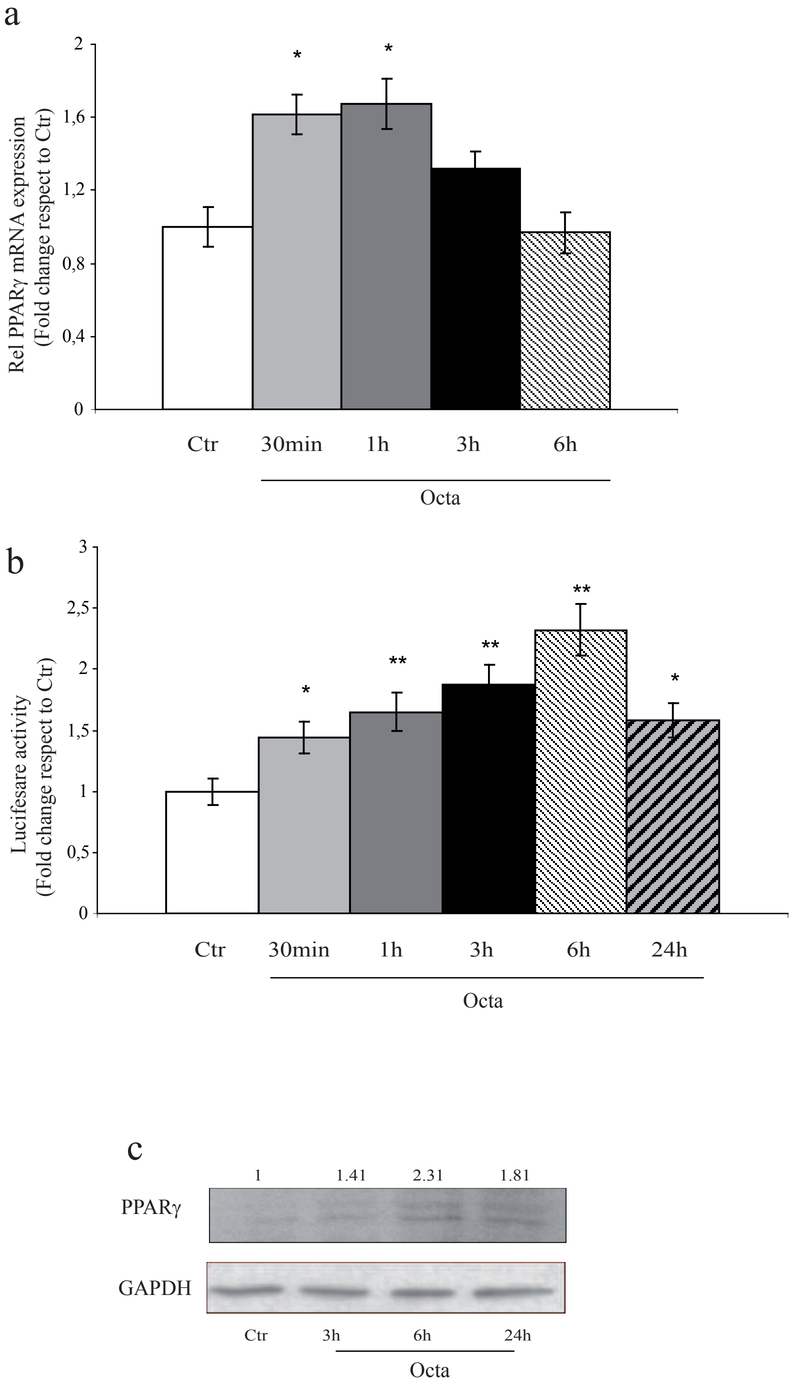

Supplementary Figure 1

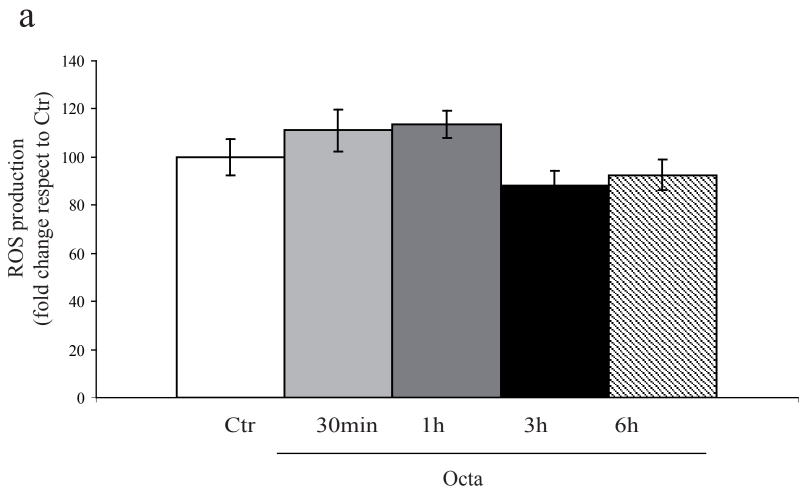

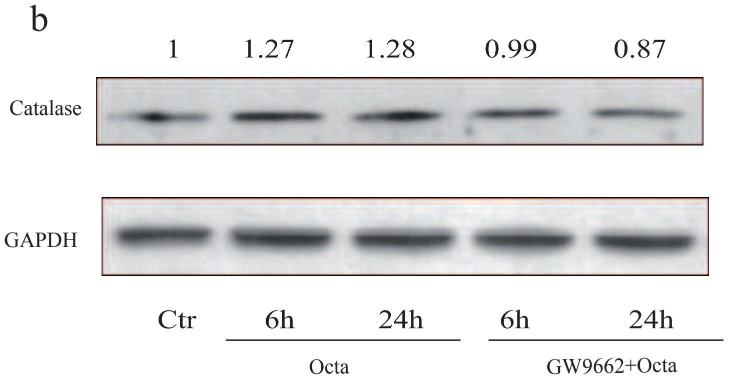

Supplementary Figure 2

a

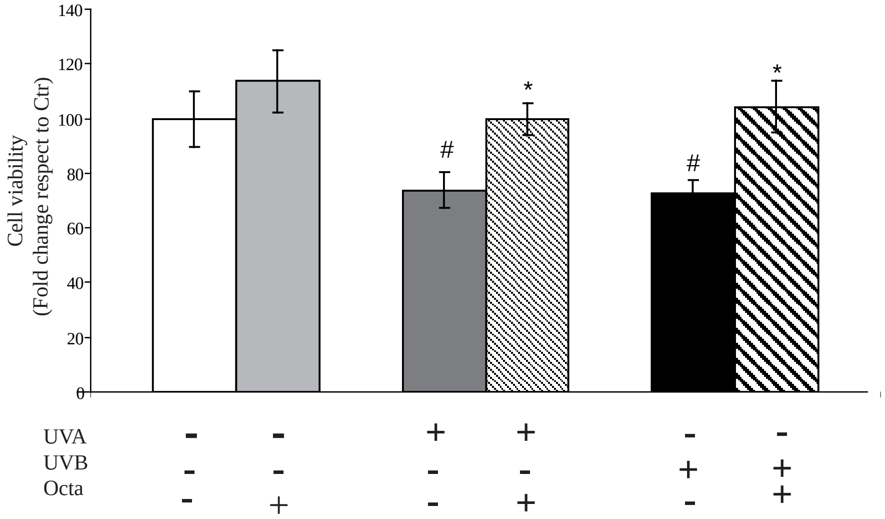

b

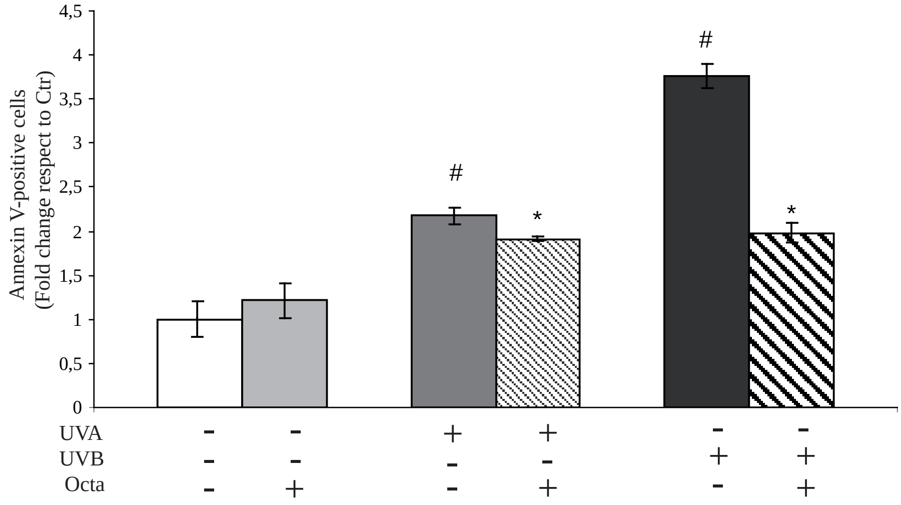

Supplementary Figure 3

Western blot, raw data Figure 1e

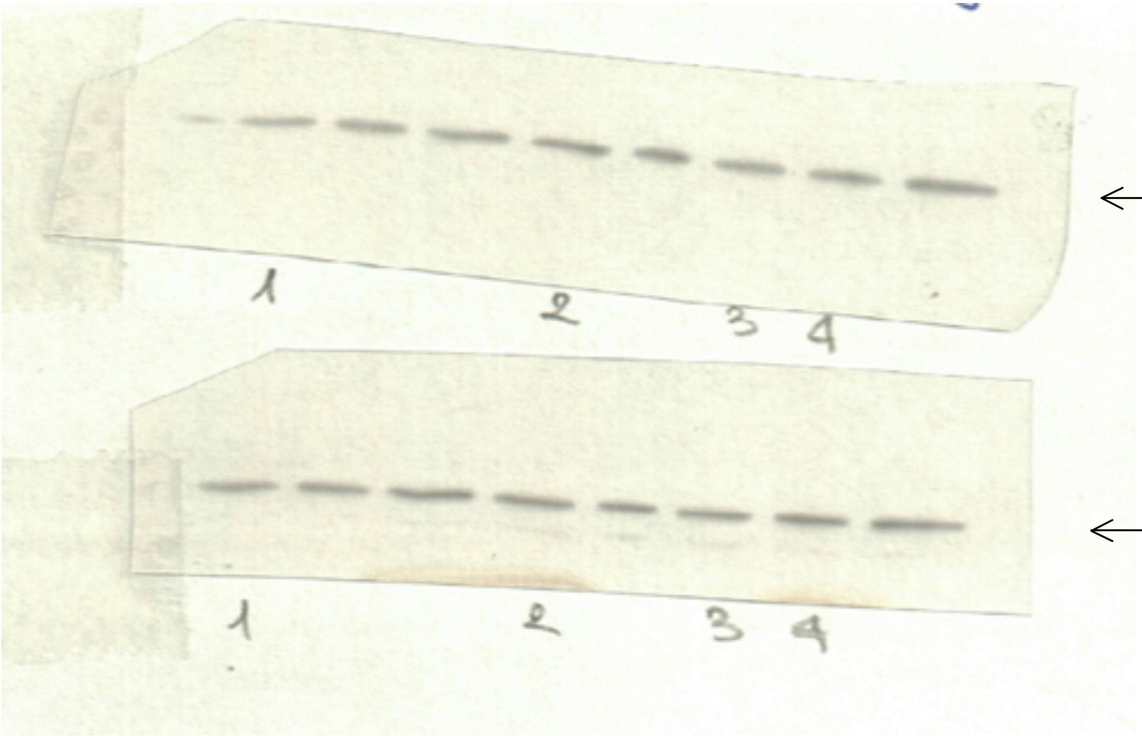

← catalase

← GAPDH

1= siCtr Ctr
2= si Ctr Octa
3= siPPARγ Ctr
4= siPPARγ Octa

Western blot, raw data Figure 2c (UVB)

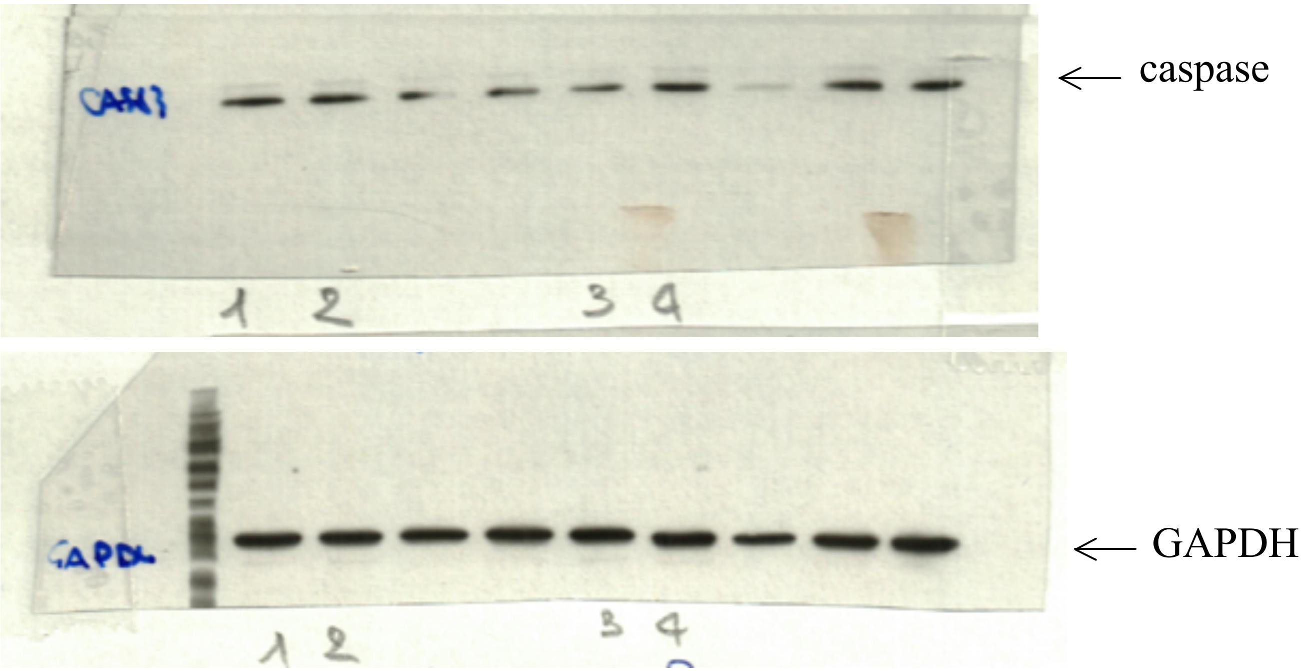

1= Ctr
2= Octa
3= UVB
4= UVB-Octa

Western blot, raw data Figure 3c and Figure 3e

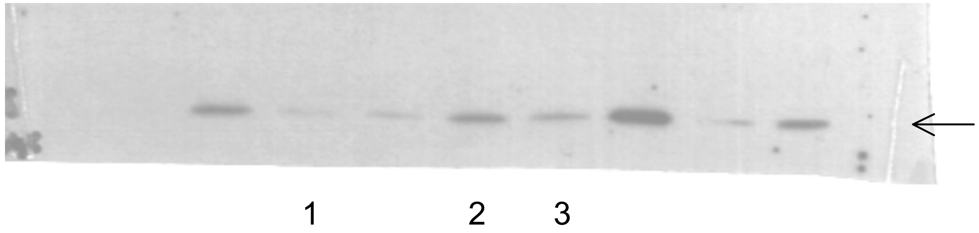

← phospho-γH2AX

1= Ctr
2= UVA
3= UVA-Octa

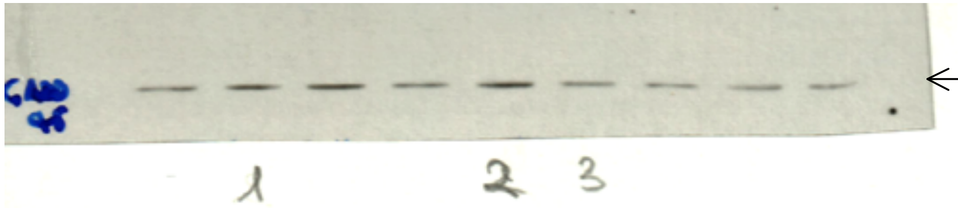

← GADD45a

1= Ctr
2= UVB
3= UVB-Octa

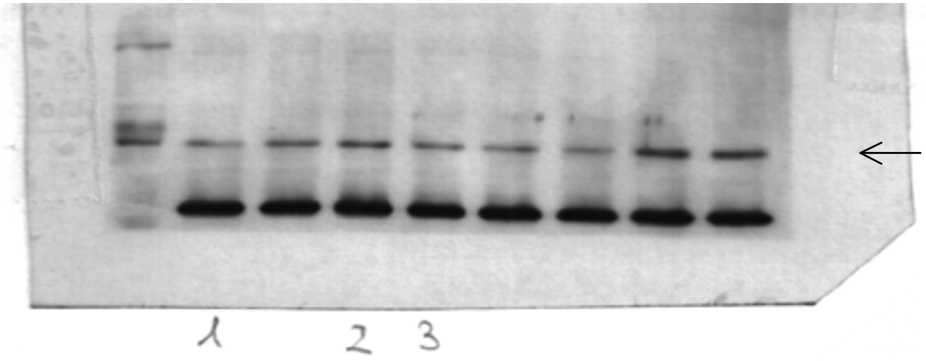

← p53

1= siCtr Ctr
2= siCtr UVB
3= siCtr UVB-Octa

Western blot, raw data Figure 4b

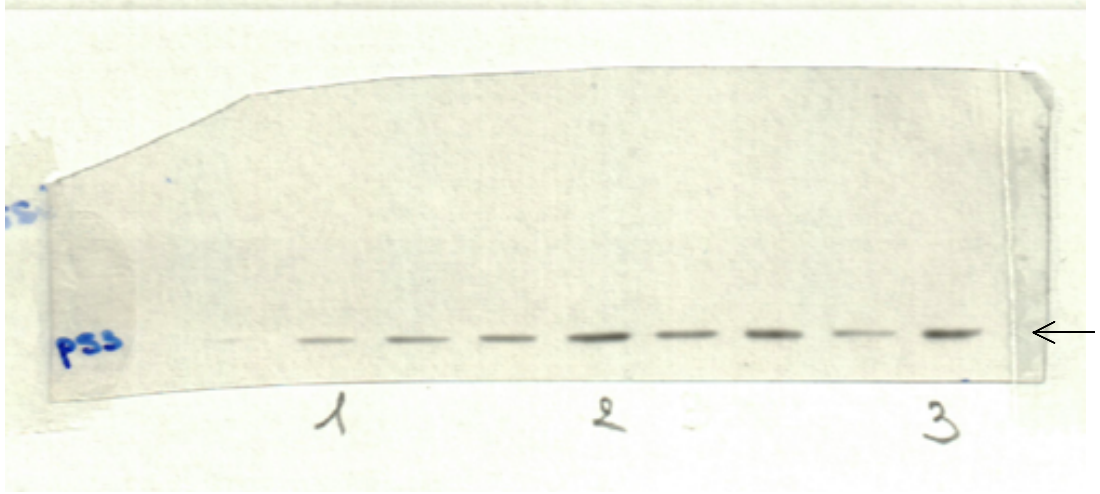

← p53

phosphoATM⟶

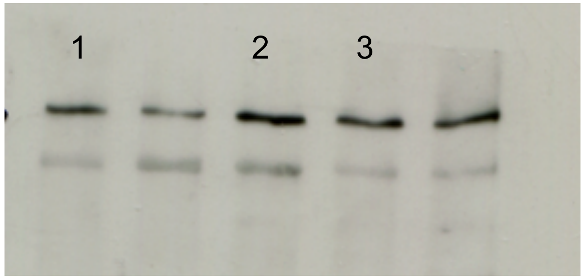

HSP70 ⟶

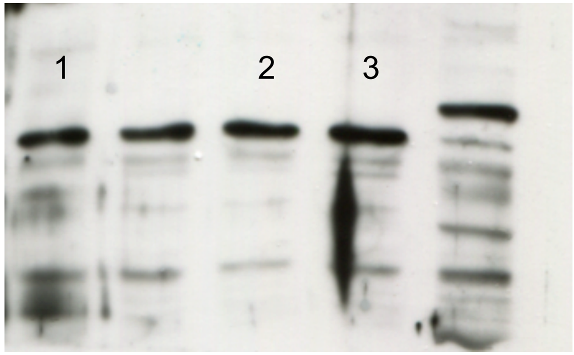

1= Ctr
2= UVB
3= UVB-Octa
